# Dextrose to Promote Granulation of Wounds

**Published:** 1913-04

**Authors:** 


Dextrose to Promote Granulation of Wounds. Alex. Cocherel, *Gaz. des Practiciens*, January 15, 1913, employs 40 per cent. solutions in normal salt and also a dry powder. The action is marked in fresh aseptic and old cicatrizing wounds, but is not manifest in septic wounds until the bacterial process has been overcome by repeated washing with sterile salt solution or even antiseptics. Oiled silk dressings are used over the powder. How he obtains a dry powdered glucose is not explained.

